# The Impact of Nonalcoholic Fatty Liver Disease on Renal Function in Children with Overweight/Obesity

**DOI:** 10.3390/ijms17081218

**Published:** 2016-07-27

**Authors:** Lucia Pacifico, Enea Bonci, Gian Marco Andreoli, Michele Di Martino, Alessia Gallozzi, Ester De Luca, Claudio Chiesa

**Affiliations:** 1 Policlinico Umberto I Hospital, Sapienza University of Rome, Viale Regina Elena 324, 00161 Rome, Italy; gianmarcoandreoli@gmail.com (G.M.A.); alessia.gallozzi@gmail.com (A.G.); esterdeluca91@libero.it (E.D.L.); 2 Department of Experimental Medicine, Sapienza University of Rome, Viale Regina Elena 324, 00161 Rome, Italy; enea.bonci@uniroma1.it; 3 Department of Radiological Sciences, Sapienza University of Rome, Viale Regina Elena 324, 00161 Rome, Italy; micdimartino@hotmail.it; 4 Institute of Translational Pharmacology, National Research Council, Via Fosso del Cavaliere 100, 00133 Rome, Italy; claudio.chiesa@ift.cnr.it

**Keywords:** nonalcoholic fatty liver disease, renal function, obesity, children

## Abstract

The association between nonalcoholic fatty liver disease (NAFLD) and chronic kidney disease has attracted interest and attention over recent years. However, no data are available in children. We determined whether children with NAFLD show signs of renal functional alterations, as determined by estimated glomerular filtration rate (eGFR) and urinary albumin excretion. We studied 596 children with overweight/obesity, 268 with NAFLD (hepatic fat fraction ≥5% on magnetic resonance imaging) and 328 without NAFLD, and 130 healthy normal-weight controls. Decreased GFR was defined as eGFR < 90 mL/min/1.73 m^2^. Abnormal albuminuria was defined as urinary excretion of ≥30 mg/24 h of albumin. A greater prevalence of eGFR < 90 mL/min/1.73 m^2^ was observed in patients with NAFLD compared to those without liver involvement and healthy subjects (17.5% vs. 6.7% vs. 0.77%; *p* < 0.0001). The proportion of children with abnormal albuminuria was also higher in the NAFLD group compared to those without NAFLD, and controls (9.3% vs. 4.0% vs. 0; *p* < 0.0001). Multivariate logistic regression analysis revealed that NAFLD was associated with decreased eGFR and/or microalbuminuria (odds ratio, 2.54 (confidence interval, 1.16–5.57); *p* < 0.05) independently of anthropometric and clinical variables. Children with NAFLD are at risk for early renal dysfunction. Recognition of this abnormality in the young may help to prevent the ongoing development of the disease.

## 1. Introduction

Concurrent with the epidemic of obesity across the world, nonalcoholic fatty liver disease (NAFLD) is becoming one of the most prevalent chronic liver disorders in both adults and children. It is now known that NAFLD is not only a risk factor for hepatic failure and hepatic carcinoma, but it is also associated with a spectrum of extrahepatic diseases generally linked to metabolic syndrome (MetS) such as type 2 diabetes, and cardiovascular disease [1,2]. Recent studies in the pediatric obese population have demonstrated that the prevalence of prediabetes and MetS is significantly increased in subjects with increased hepatic fat content, and that liver steatosis, independently of visceral and intramyocellular lipid content, is a key determinant of the impairment of liver, muscle, and adipose insulin sensitivity [3,4]. Several studies have reported associations between NAFLD and subclinical atherosclerosis and between NAFLD and cardiac function alterations, independently of established risk factors [5,6,7]. In addition, emerging evidence suggests that subjects with NAFLD have an increased risk of chronic kidney disease (CKD), defined by a decline in the estimated glomerular filtration rate (eGFR) and/or microalbuminuria and/or overt proteinuria [8,9,10,11,12]. However, no data are available in children regarding a possible association between NAFLD and impaired renal function. Recognition of the influence of NAFLD on renal function in the early age would enable us to better understand the association of NAFLD and CKD, since there is less potential for confusion with adult-onset complications.

Thus, in this study we sought to determine whether children with overweight/obesity and NAFLD show signs of renal functional alterations, as assessed by eGFR and urinary albumin excretion, compared to children with overweight/obesity but without NAFLD as well as to healthy normal-weight controls.

## 2. Results

### 2.1. Clinical and Laboratory Data from the Study Population

Clinical and laboratory data from the study population are presented in Table 1. None of the enrollees had type 2 diabetes mellitus. Patients with NAFLD were on average older than those without NAFLD and healthy controls, and had higher waist circumference (WC) as well as higher values for systolic and diastolic blood pressure (BP), higher triglycerides, aspartate aminotransferase (AST), alanine aminotransferase (ALT), uric acid, fasting glucose, insulin levels and homeostasis model assessment of insulin resistance (HOMA-IR) values, and lower high-density lipoprotein-cholesterol (HDL-C) concentrations. Patients with NAFLD had significantly lower whole-body insulin sensitivity index (WBISI) than those without NAFLD. Obese children with NAFLD and obese subjects without NAFLD had significantly higher eGFR compared to healthy controls (median, 115 (interquartile range, 104–134) and 115 (96–132) vs. 108 (100–118) mL/min/1.73 m^2^; *p* < 0.0001), whereas no differences were found between patients with and without NAFLD. However, a greater frequency of reduced eGFR (<90 mL/min/1.73 m^2^) was observed in obese subjects with NAFLD compared to obese children without liver involvement and healthy controls (17.5% vs. 6.7% vs. 0.77%, respectively; *p* < 0.0001). The proportion of children with microalbuminuria was also higher in the NAFLD group compared to obese children without liver involvement and healthy controls (9.3% vs. 4.0% vs. 0; *p* < 0.0001). None of the participants had eGFR < 60 mL/min/1.73 m^2^ or macroalbuminuria. Compared to healthy controls, the prevalence of hyperfiltration was higher in the obese cohort, regardless of liver involvement (Table 1).

To analyze the variables associated with decreased eGFR and/or microalbuminuria, we performed a logistic regression analysis in the cohort of subjects with overweight/obesity. NAFLD (odds ratio (OR), 2.34; 95% confidence interval (CI), 1.31–4.16; *p* < 0.01) was associated with abnormal renal function independently of age, gender, and pubertal status. After further adjustment for body mass index-standard deviation (BMI-SD) score, WC, hypertension, low HDL-C values, elevated triglycerides, and glucose impairment, results did not substantially change (Table 2).

### 2.2. Findings in Children with Biopsy-Proven Nonalcoholic Fatty Liver Disease (NAFLD)

To investigate the association of renal dysfunction further with advanced stages of NAFLD such as steatohepatitis (NASH), we analysed the data obtained in the small subgroup of 41 patients who underwent liver biopsy. Definite-NASH was diagnosed in 26 (63.4%) children, while not-NASH in 15 (36.5%). Compared to children without NASH, those with NASH had significantly lower eGFR (median, 88 (83–107) vs. 123 (110–130) mL/min/1.73 m^2^; *p* < 0.01). In addition, more children with NASH had eGFR of <90 mL/min/1.73 m^2^ and/or microalbuminuria than those without NASH (17/26 (65.4%) vs. 6/15 (40.0%); *p* < 0.01).

## 3. Discussion

Early recognition of impaired renal function, in particular reduced GFR, is crucial to prevent serious complications [13]. Large epidemiologic studies have found a robust relationship between obesity and risk for CKD [14,15,16]. In a community-based sample of 2585 adult individuals with renal disease at baseline and a mean follow-up of 18.5 years, BMI was reported to determine a significant increase in the odds of developing kidney disease by 23% (OR, 1.23; 95% CI, 1.08–1.41) per standard deviation unit [14]. In 9685 adults participating to the Hypertension Detection and Follow-Up Program, free of CKD at baseline, the incidence of CKD was 28%, 31%, and 34%, respectively, in the ideal body mass index, overweight, and obese groups, after a follow-up of five years [15]. After adjustment for variables, such as age, gender, race, diabetes mellitus, mean baseline diastolic BP, and slope of diastolic BP, at baseline both overweight (OR, 1.21; 95% CI, 1.05 to 1.41) and obesity (OR, 1.40; 95% CI, 1.20 to 1.63) were associated with increased incident CKD odds at year 5 [15]. In addition, a retrospective cohort study of 320,252 adults, who were followed for 15 to 35 years, showed that a high BMI (≥25.0 kg/m^2^) determined who is at high risk of developing end-stage renal disease [16]. Taken together, these studies indicate that higher BMI in adults is a risk factor for the development of new onset kidney disease. Several possible pathophysiologic pathways may underlie this association. One possibility is that particular characteristics of obesity may account for the association between obesity and CKD. Indeed, obesity constitutes a complex syndrome involving metabolic traits and other factors that may interact with other environmental factors, leading to an increased risk for developing kidney disease. Clustering of these traits defines MetS, which has been reported to be consistently associated with CKD in cross-sectional studies [17,18].

NAFLD has been recently found to be an additional feature of MetS, with the main underlying cardiometabolic risk factors of the syndrome being abdominal obesity and insulin resistance [19,20]. Of note, insulin resistance is not only a metabolic determinant for the development of NAFLD but is also a predictor of incident CKD [21,22]. In addition, atherogenic dyslipidemia and type 2 diabetes are established risk factors for CKD [23,24]. As a consequence, many authors have concluded that NAFLD may have a pathogenic role in the development of CKD. The results of a recent meta-analysis have shown that (1) there is a positive relationship between NAFLD and an increased risk of CKD in adults; (2) the severity of liver disease is associated with an increased risk and severity of CKD; and (3) these relationships are maintained even after taking account of the well-known risk factors for CKD, and are independent of whole body/abdominal obesity and insulin resistance [8].

In our study, we investigated the influence of NAFLD on kidney function in a large pediatric population. This is the first study to demonstrate that overweight/obese children with NAFLD have a greater frequency of eGFR of <90 mL/min/1.73 m^2^ as well as of microalbuminuria than overweight/obese children without NAFLD. Furthermore, in the small number of children with biopsy-proven NAFLD we were able to show that the decline in renal function was greater in those with NASH. It is important to point out that subjects with obesity represent a particular population in whom early renal lesion consists of hyperfiltration. In fact, in line with previous studies [25,26,27], one of the main findings of this study was that children with overweight/obesity compared to normal-weight subjects had a higher prevalence of hyperfiltration, regardless of liver involvement. Glomerular hyperfiltration is well-recognized as an early renal injury occurring in a number of clinical conditions, including diabetes, hypertension, and obesity [28]. Hyperfiltration is hypothesized to be a precursor of intraglomerular hypertension responsible for albuminuria. GFR then declines progressively as albuminuria increases which may cause, in the long run, end-stage renal failure [28]. Thus, in obese patients with NAFLD, we should pay attention for minor impairment on renal function, since hyperfiltration may mask a pathological decline in renal function.

The most plausible explanation for our findings is that the renal abnormalities in overweight/obese children with NAFLD may reflect the coexistence of underlying metabolic risk factors including higher BP, more dyslipidemia, and more insulin resistance compared to children without liver involvement. However, because in our study the presence of NAFLD remained significantly associated with decreased eGFR and/or microalbuminuria after taking account of traditional metabolic traits, we cannot rule out the possibility that NAFLD might at least in part contribute to the development of renal dysfunction independently of shared cardiometabolic risk factors.

The strength of our study includes a large sample size and an extensive and complete analysis of metabolic variables. Nonetheless, some limitations require consideration. First, the cross-sectional design of the study precludes the establishment of causal relationship between NAFLD and abnormal kidney function. Second, we used an estimated GFR instead of a directly measured GFR to define renal function. The gold standard technique is clearance of inulin, but practical problems limit the application of this cumbersome methodology in children because of the necessity for steady-state infusion, and a urine sampling with a bladder catheter. Other tests for determining GFR are clearance of alternative exogenous markers such as iothalamate, which are also complex and difficult to do in routine clinical practice. Recent studies in children have reported current eGFR creatinine- and/or cystatin C-based equations to be reliable methods to assess kidney function, with some variations depending on the GFR ranges and the BMI classes [29,30,31]. The updated Schwartz formula has been shown to be accurate for estimating GFR when compared to inulin clearance as well as to iothalamate clearance in children and adolescents, with a wide range of renal function [29,30]. Moreover, obesity has not been found to affect GFR as estimated by Schwartz formula [31]. Finally, we measured creatinine concentration by kinetic colorimetric compensated technique, whereas in the updated Schwartz formula, it was determined by an enzymatic method. The two methods, however, are highly correlated [29].

In conclusion, our present study suggests that obese children with NAFLD are at risk for early renal dysfunction. Recognition of this abnormality in the young may be important because treatment to reverse the process is most likely to be effective if applied earlier in the disease process.

## 4. Materials and Methods

### 4.1. Study Subjects

This observational cross-sectional study included 596 children and adolescents with overweight/obesity who were consecutively recruited at the outpatient Clinics (Hepatology, Lipid and Nutrition) of the Department of Pediatrics, Sapienza University of Rome, Italy, between 2007 and 2015. Two hundred and sixty eight subjects met the criteria for the diagnosis of NAFLD (i.e., hepatic fat fraction (HFF) ≥5% on magnetic resonance imaging (MRI)) [32]. In all enrollees, hepatic virus infections (hepatitis A–E and G, cytomegalovirus, and Epstein–Barr virus), autoimmune hepatitis, metabolic liver disease, α-1-antitrypsin deficiency, cystic fibrosis, Wilson’s disease, hemochromatosis, and celiac disease were excluded using appropriate tests [6,7]. In 41 of the NAFLD patients, due to persistent elevations in ALT concentrations, a liver biopsy was performed. The other 328 participants had HFF < 5% on MRI, normal levels of aminotransferases, and no evidence of chronic liver diseases (see above). Use of hepatotoxic drugs, as well as a history of type 1 or 2 diabetes, smoking and chronic alcohol intake were also exclusion criteria. None of the subjects had a history or known clinical, laboratory, and imaging signs of renal disease.

The study also included a total of 130 apparently healthy normal-weight school students drawn from four randomly selected schools in the Rome area. All students were invited to take part in a pilot study whose objective was the prevention of cardiovascular disease in childhood. Eligibility criteria included age- and gender-specific BMI; no history of renal and liver diseases as well as of alcohol consumption and smoking; normal liver ultrasound, and normal biochemical values.

All study subjects had a complete physical examination, as reported in detail elsewhere [5,6]. The degree of obesity was quantified using Cole’s least mean-square method, which normalizes the skewed distribution of BMI and expresses BMI as SD score [33].

The study protocol was reviewed and approved by the Ethics Committee of Policlinico Umberto I Hospital, Rome, Italy. Written informed consent was obtained from the parents, or guardians of the children included in this study, in accordance with principles of Helsinki Declaration.

### 4.2. Laboratory Mmeasurements

Blood samples were taken from all study subjects, after an overnight fast, for estimation of glucose, insulin, urea nitrogen, creatinine, uric acid, total cholesterol, HDL-C, triglycerides, ALT, AST, and gamma-glutamyl transferase. An oral glucose tolerance test was performed for all overweight/obese children using 1.75 g/kg of glucose up to a maximum of 75 g. Two-hour post-load glucose and insulin were analyzed. Insulin resistance was calculated by the HOMA-IR. Insulin sensitivity was calculated by the WBISI with reduced time points according to the following formula: 10,000/√ (fasting glucose × fasting insulin × 2 h post-load glucose × 2 h post-load insulin) [34].

All analyses were performed on COBAS 6000 (Roche Diagnostics, Risch-Rotkreuz, Switzerland). Creatinine concentrations were measured by the kinetic colorimetric compensated Jaffé method using the Roche platform and the CREJ2–creatinine Jaffé Gen.2 assay (Roche Diagnostics, Identification number, 0769282), which was isotope-dilution mass spectrometry standardized, traceable to National Institute of Standards and Technology creatinine standard reference material (SRM 914 and SRM 967). Urinary albumin was determined on 24 h urine collections by the turbidimetric immunoassay ALBT2 (Roche Diagnostics, Identification number, 0767433).

eGFR was calculated using the updated Schwartz formula: 0.413 × height (cm)/serum creatinine (mg/dL) [35].

### 4.3. Liver Ultrasound Eexamination and Magnetic Resonance Imaging

Liver ultrasound was performed by a single operator. Hepatic steatosis was diagnosed on the basis of the following features: a diffuse increase in echogenicity (a bright liver), liver to kidney contrast, deep beam attenuation, vascular blurring, and loss of definition of the diaphragm [36]. The amount of HFF was measured by MRI using the two-point Dixon method as modified by Fishbein [37], as previously described and validated [32,38].

### 4.4. Liver Biopsy

Liver biopsy was performed in 41 subjects because of persistent elevation in ALT. The clinical indication for biopsy was either to assess the presence of nonalcoholic steatohepatitis (NASH) or to determine the presence of other independent or competing liver diseases. The main histologic features of NAFLD were scored using the NASH Clinical Research Network criteria [39]. Biopsies were categorized into not-NASH and definite-NASH.

### 4.5. Definitions

Overweight and obesity were defined according to age- and gender-specific cut-off points of BMI defined by the International Obesity Task Force criteria as proposed by Cole et al. [33]. Elevated BP was defined as systolic or diastolic BP ≥ 90th percentile for age, gender, and height [40]. Impaired fasting glucose was defined as glucose ≥5.6 mmol/L. High waist circumference (WC), high triglycerides, and low HDL-C were defined using the cut-off proposed by Cook et al. [41]. Insulin resistance was defined by 90th percentile of HOMA-IR for age and gender in our population of healthy normal-weight children. Abnormal albuminuria was defined as a 24-h urinary albumin excretion rate ≥30 mg (i.e., microalbuminuria was diagnosed if the 24-h albumin excretion rate was 30–299 mg and macroalbuminuria if the 24-h albumin excretion rate was ≥300 mg) [42]. As recommended by Kidney Disease Improving Global Outcomes (KDIGO) guidelines, eGFR categories were classified as follows: normal or high ≥90 mL/min/1.73 m^2^; mildly decreased, 60–89; mildly to moderately decreased, 45–59; moderately to severely decreased, 30–44; severely decreased, 15–29; and kidney failure <15 [42]. In the absence of an agreement in the literature, we defined glomerular hyperfiltration as eGFR > 95th percentile of that observed in our population of healthy normal-weight subjects (i.e., eGFR > 139 mL/min/1.73 m^2^).

### 4.6. Statistical Analysis

Statistical analyses were performed using the SPSS package (version 22.0, SPSS Inc., Chicago, IL, USA). Data are reported as means and standard deviations for normally distributed variables, or as median and interquartile range for non-normally distributed variables. Differences between study groups in quantitative variables were evaluated by one-way analysis of variance (ANOVA) or Kruskal–Wallis test, as appropriate. Proportions were compared by the chi square test. Logistic regression analysis was used to assess the independent association of NAFLD with abnormal kidney function, after adjustment for age, gender, pubertal status, BMI-SD score, WC, hypertension, low HDL-C values, elevated triglycerides, and glucose impairment.

## Figures and Tables

**Table 1:** Clinical and laboratory characteristics of the study population.

	Normal Weight	NO NAFLD	NAFLD	*p* Value *
No. patients	130	328	268	<0.0001
Age, years	10.6 (3.5)	10.1 (2.9)	11.2 (2.9) ^d^	<0.0001
Male sex, *n* (%)	61 (46.9)	151 (46.0)	166 (61.9) ^a,d^	<0.0001
BMI-SD score	0.17 (0.85)	1.85 (0.45) ^a^	2.0 (0.45) ^a,d^	<0.0001
Waist circumference, cm	65 (10)	82 (12) ^a^	92 (13) ^a,d^	<0.0001
Systolic BP, mmHg	102 (11)	107 (12) ^b^	114 (12) ^a,d^	<0.0001
Diastolic BP, mmHg	63 (7)	65 (9) ^c^	69 (8) ^a,d^	<0.0001
Total cholesterol, mg/dL	166 (145–186)	161 (139–187)	159 (137–181)	0.077
LDL-C	92 (72–118)	94 (76–115)	94 (74–111)	0.78
HDL-C, mg/dL	56 (50–83)	51 (44–60) ^a^	46 (38–53) ^a,d^	<0.0001
Triglycerides, mg/dL	62 (50–83)	70 (50–99)	89 (58–127) ^a,d^	<0.0001
AST, U/L	22 (20–30)	23 (20–27) ^c^	26 (21–35) ^a,d^	<0.0001
ALT, U/L	16 (13–20)	18 (14–23) ^b^	31 (19–54) ^a,d^	<0.0001
Uric acid	0.21 (0.18–0.25)	0.25 (0.22–0.29) ^a^	0.28 (0.24–0.34) ^a,d^	<0.0001
Glucose, mg/dL	83 (7)	83 (7)	85 (11)	0.002
Insulin, μU/mL	7.5 (4.3–10.5)	11.1 (7.5–15.4) ^a^	15.2 (10.1–23.2) ^a,d^	<0.0001
HOMA-IR	1.58 (0.90–2.20)	2.30 (1.55–3.22) ^a^	3.23 (2.05–5.0) ^a,d^	<0.0001
WBISI	-	6.5 (4.5–9.0)	3.5 (2.4–5.6) ^d^	-
eGFR, mL/min/1.73 m^2^	108 (100–118)	115 (104–134) ^a^	115 (96–132) ^a^	<0.0001
eGFR < 90 mL/min/1.73 m^2^, *n* (%)	1 (0.77)	22 (6.7) ^b^	47 (17.5) ^a,d^	<0.0001
eGFR > 139 mL/min/1.73 m^2^, *n* (%)	6 (4.6)	56 (17.0) ^a^	46 (17.2) ^a^	0.002
Microalbuminuria, *n* (%)	0	13 (4.0) ^a^	25 (9.3) ^a,d^	<0.0001

Results are expressed as *n* (%), mean (standard devation), or median (interquartile ranges). * Anova or Kruskal-Wallis test; ^a^ *p* < 0.0001; ^b^ *p* < 0.01; ^c^ *p* < 0.05 vs. controls; ^d^ *p* < 0.0001 vs. obese children without NAFLD; NAFLD, nonalcoholic fatty liver disease; BMI-SD score, Body mass index- standard deviation score; BP, Blood pressure; LDL-C, Low density lipoprotein-cholesterol; HDL-C, High-density lipoprotein-cholesterol; AST, Aspartate aminotransferase; ALT, Alanine aminotransferase; HOMA-IR, Homeostasis model assessment of insulin resistance; WBISI, Whole-body insulin sensitivity index; eGFR, estimated glomerular filtration rate.

**Table 2:** Associations of NAFLD with eGFR < 90 mL/min/1.73 m^2^ and/or microalbuminuria in children with overweight/obesity.

Variables	Odds Ratio (95% CI)	*p* Value
Adjusted model 1: age, gender, pubertal status	2.34 (1.31–4.16)	0.004
Adjusted model 2: model 1 plus BMI-SD score, WC, High BP, High TG, low HDL-C, and high FG	2.54 (1.16–5.57)	0.02
Adjusted model 3: model 1 plus BMI-SD score, WC, High BP, High TG, low HDL-C, and IR	2.30 (1.02–5.17)	0.04

CI, confidence interval; eGFR, estimated glomerular filtration rate; BMI-SD score, Body mass index- standard deviation score; WC, waist circumference; BP, Blood pressure; TG, triglycerides; HDL-C, High-density lipoprotein-cholesterol; FG, fasting glucose; IR, insulin resistance.
